# Changes in the Distribution of Intrauterine Microbiota May Attribute to Immune Imbalance in the CBA/J×DBA/2 Abortion-Prone Mice Model

**DOI:** 10.3389/fimmu.2021.641281

**Published:** 2021-03-08

**Authors:** Shiyu Bai, Bingqian Huang, Shuai Fu, Menglan Zhu, Lihao Hu, Liqiong Zhu, Manqi Chen, Zicheng Zhang, Jianping Tan, Jianping Zhang, Hui Chen

**Affiliations:** ^1^Department of Obstetrics and Gynecology, Sun Yat-sen Memorial Hospital, Sun Yat-sen University, Guangzhou, China; ^2^Center for Reproductive Genetics and Reproductive Medicine, Sun Yat-sen Memorial Hospital, Sun Yat-sen University, Guangzhou, China; ^3^Department of Radiation Oncology, Shenzhen Traditional Chinese Medicine Hospital, The Fourth Clinical Medical College of Guangzhou University of Chinese Medicine, Shenzhen, China

**Keywords:** microbiota, 16S rRNA sequencing, abortion-prone model, immune imbalance, maternal-fetal interface

## Abstract

**Background:** Female Genital Tract (FGT) is an important micro-ecological area of human body. Microbiota in the lower reproductive tract may subsequently invade the uterine cavity during embryo implantation and produce immune responses. CBA/J×DBA/2 mating combination has been widely used as an abortion-prone mice model but whether microbiota existed in their uterine cavity remains unclear. In this context, the role of the microbial communities in immune response deserves attention.

**Objective:** To investigate the relationship between the distribution of microbiota in the uterine cavity of CBA/J×DBA/2 abortion-prone mouse model and the immune imbalance of the maternal-fetal interface.

**Methods:** In this study, female CBA/J mice were paired with male DBA/2 mice to develop an abortion-prone model (BA group), and with male BALB/c mice to build a standard pregnancy model (BC group). The non-pregnant female mice were served as the control group (C group). Uterine flushing fluid and sera were collected on day 13.5 of pregnancy. 16S rRNA sequencing technology was used to analyze the distribution of intrauterine microbiota. Phylogenetic Investigation of Communities were conducted to predict the microbiota functions by Reconstruction of Unobserved States (PICRUST) and Kyoto Encyclopedia of Genes and Genomes (KEGG). The serum IL 10, INF-γ, and TNF-α levels were examined using Enzyme-linked immunosorbent assay (ELISA) method.

**Results:** All samples were detected with microbial communities. The α diversity (*p* = 0.00077) had significant differences among three groups. *Proteobacteria* was the most dominant phylum in C group (mean = 83.21%) and BA group (mean = 43.23%). *Firmicutes* was dominant in BC group (mean = 46.4%), as well as the second dominant one in C group (mean = 12.63%) and BA group (mean = 40.55%). Microbiota functions were associated with metabolism and immune response through the NOD-like receptor signaling pathway. The serum IL 10 level in BA group were significantly lower than that in BC group (10.14 ± 1.90 pg/ml, *n* = 8; vs. 19.03 ± 1.82 pg/ml, *n* = 10; *p* = 0.004). The serum TNF-α and INF-γ level in BA group were also significantly higher than that in BC group (523.1 ± 58.14 pg/ml, *n* = 8 vs. 310.3 ± 28.51 pg/ml, *n* = 10, *p* = 0.0029; 69.22 ± 5.38 pg/ml, *n* = 8 vs. 50.85 ± 2.45 pg/ml, *n* = 10, *p* = 0.0042).

**Conclusion:** Microbial communities were colonized in uterine cavity of CBA/J mice both at non-pregnant stage and pregnant stage when mated with both BALB/c and DBA/2 male mice. The differentially abundant microbiome may be attributed to the immune tolerance through binding to the NOD-like receptor.

## Introduction

Maintaining pregnancy requires an immune balance microenvironment at the maternal-fetal interface. Innate immunity and acquired immunity jointly contribute to this immune tolerance microenvironment, while an imbalanced stage could lead to pregnancy loss (1). The surviving embryos are prone to placental related diseases like intrauterine fetal growth restriction and hypertension during pregnancy (2). Many factors may break the immune balance and microbial invasion that could activate the innate immunity. The Female Genital Tract (FGT) is an important micro-ecological area in human body, hosting many microorganisms and closely linked to human health. Traditionally, uterine cavity has been often known as a classic sterile cavity. However, Recent studies have found a certain amount of symbiotic microbiota in the uterine cavity and some of them only found in the uterine cavity (3). Besides, microbiota from the lower reproductive tract can enter the uterine cavity and stimulate trophoblasts to produce an immune response at the stage of embryo implantation.

NLR receptor (NOD-like receptor) family, as a major cytoplasmic PPR (Pattern recognition receptors), plays an important role in the innate immune system, maintaining the intracellular environment. It plays a defensive function by identifying PAMPs (pathogen-associated molecular pattern) and endogenous DAMPs (damage-associated molecular patterns) that invade cells (4). Recent studies have shown that the degradation products of bacterial cell wall peptidoglycan (PGN) can be recognized by both NOD1 and NOD2 proteins, and the PGN motifs they recognize are distinct. The smallest structure recognized by NOD1 is γ-D-glutamic acid-meso-diaminopimelic acid (γ-D-glu-meso-DAP, mesoDAP), which mainly exists in the PGN of G^−^bacteria. Therefore, NOD1 can specifically recognizes G^−^bacteria. The smallest structure recognized by NOD2 is MDP (muramyl dipeptide) which exists in the PGN of all bacteria, thus NOD2 is both a receptor for G^+^ bacteria and G^−^ bacteria (5). Our previous study confirmed that URSA (Unexplained Recurrent Spontaneous Abortion) patients have high expressions of NOD1 and NOD2, affecting the behavior of trophoblasts through the MAPK/p38 signaling pathway (6). The CBA/J'DBA/2 mice model has been commonly employed to investigate immune tolerance at the maternal-fetal interface with pregnancy failure. Here we aim to explore whether there is a linkage between the distribution of intrauterine microbiota and immune imbalance at the maternal-fetal interface in the CBA/J'DBA/2 abortion mice model.

## Materials and Methods

### Animals and Treatment

Ten to 12 weeks old of male DBA/2 mice, male BALB/c mice and female CBA/J mice were purchased from Beijing HFK Bioscience Co., LTD. All animals were feed at the Center for Disease Model Animals of Sun Yat-sen University and maintained under a 12-h light-dark cycle at constant temperature (20–22°C) with free access to standard food and water. Female CBA/J mice were randomly allocated into abortion model group (BA group, *n* = 8), normal pregnant control group (BC group, *n* = 10), and normal non-pregnant control group (C group, *n* = 8). Female mice and male mice were mated at 18:00 p.m. to construct a normal pregnancy model (CBA/J×BALB/c) and an abortion model (CBA/J×DBA/2). Female CBA/J mice detected vaginal plugs on day 0.5 of gestation. On day 13.5 of pregnancy, animals were killed by CO_2_ inhalation. Embryo absorption rate were measured in order to determine the accuracy of the abortion model. All animal experimental protocols were approved by the Animal Ethics Committee of Sun Yat-Sen University (Guangzhou, China).

### Sample Collection

**Figure 1:**
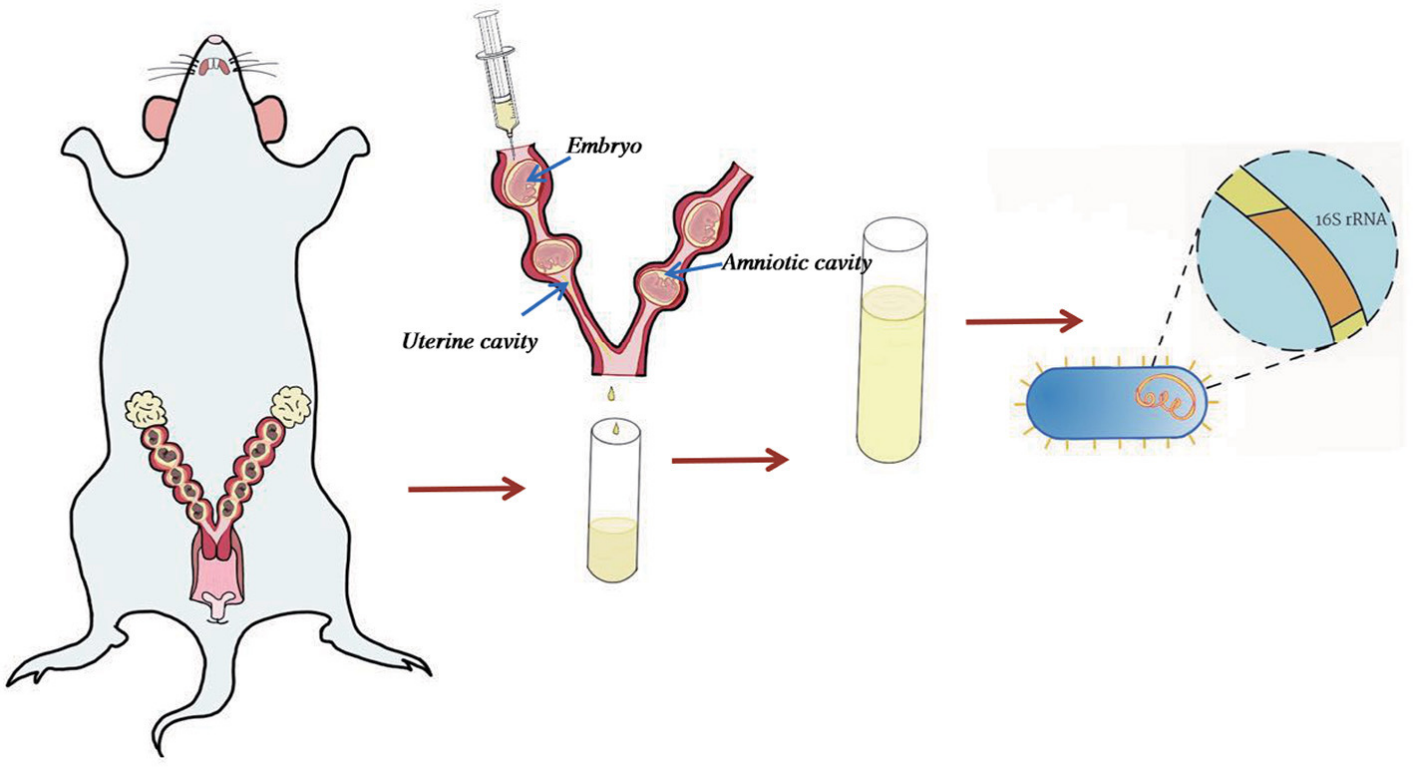
Sample collection method. Uterus was separated from the cervix and fallopian tubes. 1 ml RNase- and DNase-free water was used to rinse the uterine cavity to avoid piercing the amniotic cavity or piercing the uterine wall. The flushing fluid sample was collected for 16s rRNA sequence.

The uterus was separated from the cervix and fallopian tubes aseptically. One ml RNase- and DNase-free water was injected from one side of uterine horn and flushing fluid was collected in cryopreservation tube, placed into liquid nitrogen, and then transferred to −80°C for future analysis (Figure 1). Samples were collected under sterile conditions to avoid contamination. Puncturing into the amniotic cavity had avoided any interference with the amniotic fluid. Due to the difficulty of obtaining materials for uterine flushing fluid, we only collected 2 cases in C group, 5 cases in BA group and 5 cases in BC group.

### DNA Extraction and 16S rRNA Sequencing

Genomic DNA were extracted from the frozen uterine cavity fluid sample by QIAmp DNA mini-kit (Qiagen, USA) following the manufacturer's protocol. Sterile saline was chosen as the negative control. The V3-V4 region of the 16S rRNA gene sequencing was completed by MAGIGENE (Guangzhou, China) according to the standard service workflow. Primers 357-F (5′-ACTCCTACGGRAGGCAGCAG-3′) and 806R (5′-GGACTACHVGGGTWTCTAAT-3′) was used to amplify 16S rRNA gene by PCR following the Illumina miseq protocols. PCRs were run in duplicate using GeneAmp^®^ PCR System 9700 (California, USA) using thermocycling conditions of 94°C for 2 min, 38 cycles of 94°C for 30 s, 56°C for 30 s, 72°C for 30 s, a final extension at 72°C for 5 min, and a final temperature at 10°C. PCR products were checked by agarose gel electrophoresis and collected using AxyPrepDNA (Huangzhou, China). The PCR products were quantified using FTC-3000 TM real-time PCR and sequenced using MiSeq^®^ Reagent Kit v3 (Illumina) on a MiSeq-Illumina platform (Lifesequencing sequencing service, Valencia, Spain).

### Bioinformatics and Data Analysis

Sequences were processed through QIIME (7). Based on their sequence similarity using UPARSE, all sequences were clustered into operational taxonomic units (OTU), with a threshold of sequence similarity as 0.97. Singletons and OTUs with a relative frequency below 0.01 were removed. Alpha diversity was evaludated using QIIME and Shannon method was applied to analyze the biodiversity of samples. The X-axis represented the grouping, and the Y-axis represented the corresponding Shannon diversity index. The shape on the plots show the richness of alpha diversity that the higher Y-axis values suggested the richer species (**Figure 3A**).

Beta diversity was conducted using Principal coordinates analysis (PCoA) plots. The LEfSe method (8) was applied to analyze and identify the differentially abundant microbiome across three groups. Linear discriminant analysis (LDA) was employed to identify communities or species with significant differences in the division of samples into different groups, depending on the taxonomic composition. The total statistical analysis on bacterial taxonomic identification was performed using Calypso software (version 8.10).

### PICRUST and KEGG Enrichment

Functional Potential of the Microbiome was used PICRUST and the GO and KEGG database were adopted by putting in the OTU abundances (9).

### Enzyme-Linked Immunosorbent Assay (ELISA)

Sera were collected after centrifugation of blood obtained from the abdominal aortic puncture and stored at −80°C. The serum IL 10, INF-γ and TNF-α levels were examined using ELISA method following manufacturer's instruction.

### Data Analysis

Statistical analysis was performed using SPSS 19.0. Measurement data were expressed as mean ± standard deviation (X ± s). The mean comparisons of serum IL 10, INF-γ and TNF-α levels between two groups were performed by *t*-test. The comparison of the rate of two groups was performed using Pearson Chi-square test. Alpha diversity was analyzed using the analysis of variance (ANOVA) method. *P* < 0.05 was considered to be statistically significant.

## Results

### Abortion-Prone Model and Embryo Resorption Rate

**Figure 2:**
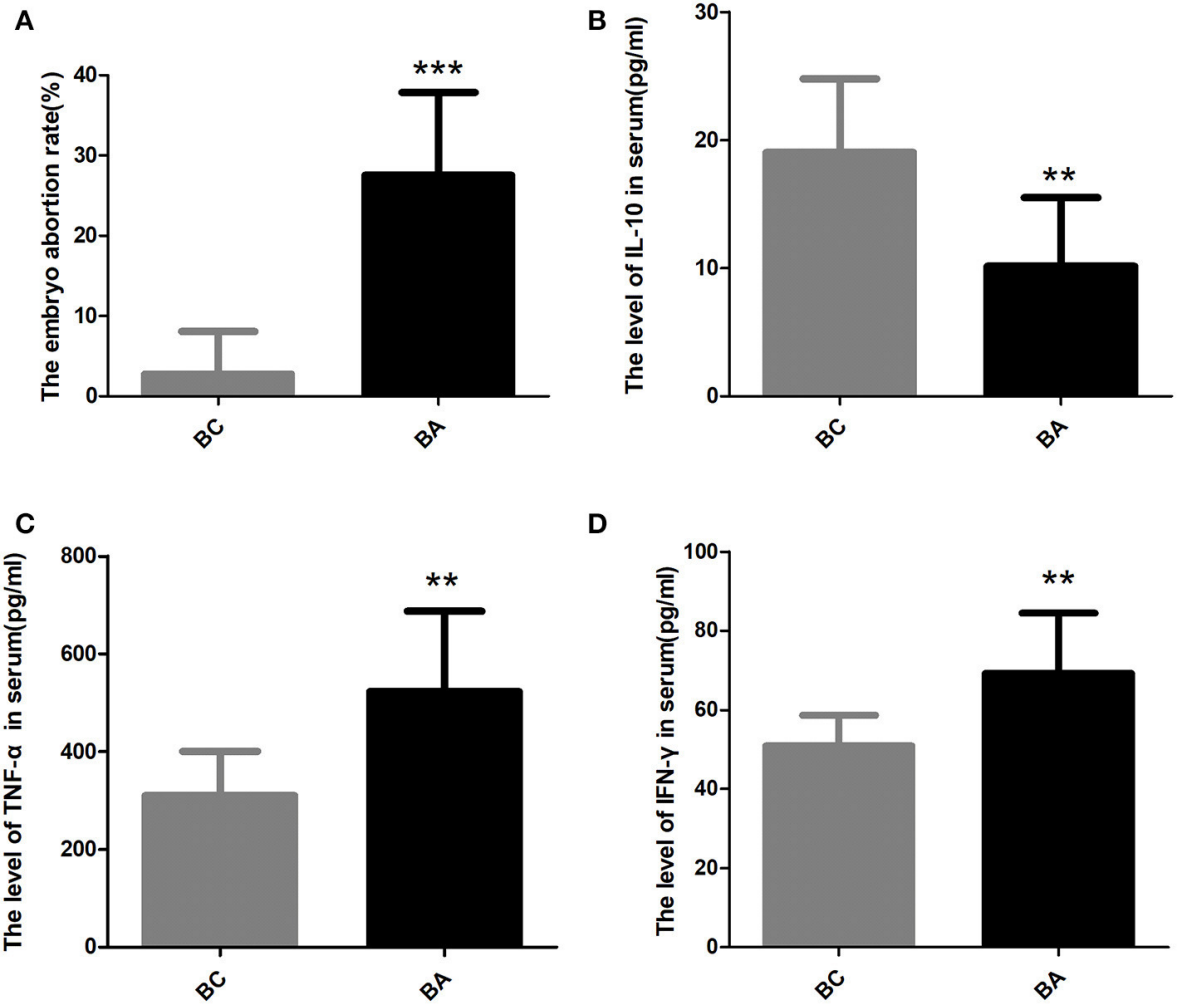
The embryo abortion rate and the level of inflammatory factors in the serum. **(A)** The embryo abortion rate in BC group was significantly lower than that in BA group (2.74 ± 1.68% vs. 27.51 ± 3.652%, *P* < 0.0001). **(B)** The serum IL-10 level in BA group were significantly lower than that in BC group (10.14 ± 1.90 pg/ml, *n* = 8; vs. 19.03 ± 1.82 pg/ml, *n* = 10; *p* = 0.004). **(C)** the serum TNF-α level in BA group (523.1 ± 58.14 pg/ml) were significantly higher than that in BC group (310.3 ± 28.51 pg/ml, *p* = 0.0029). **(D)** The serum INF-γ level in BA group (69.22 ± 5.38 pg/ml) were significantly higher than that in BC group (50.85 ± 2.45 pg/ml, *p* = 0.0042). ** indicates *P* < 0.01; *** indicates *P* < 0.001.

The embryo resorption rate of BA group was 27.51 ± 3.652%, which was significantly higher than that in BC group (2.74 ± 1.68%, *P* < 0.0001) (Figure 2A). Following our previous study, the current abortion mice model was considered successful.

### The Level of Inflammatory Factors in the Serum

The serum IL-10 level in BA group was significantly lower than that in BC group (10.14 ± 1.90 pg/ml, *n* = 8; vs. 19.03 ± 1.82 pg/ml, *n* = 10; *p* = 0.004) (Figure 2B). However, the serum TNF-α level in BA group (523.1 ± 58.14 pg/ml) were substantially higher than that in BC group (310.3 ± 28.51 pg/ml, *p* = 0.0029) (Figure 2C). The serum INF-γ level between two groups had the same trend, with the level in BA group (69.22 ± 5.38 pg/ml) significantly higher than that in BC group (50.85 ± 2.45 pg/ml, *p* = 0.0042) (Figure 2D).

### Dataset Features and Bacterial Diversity

**Figure 3:**
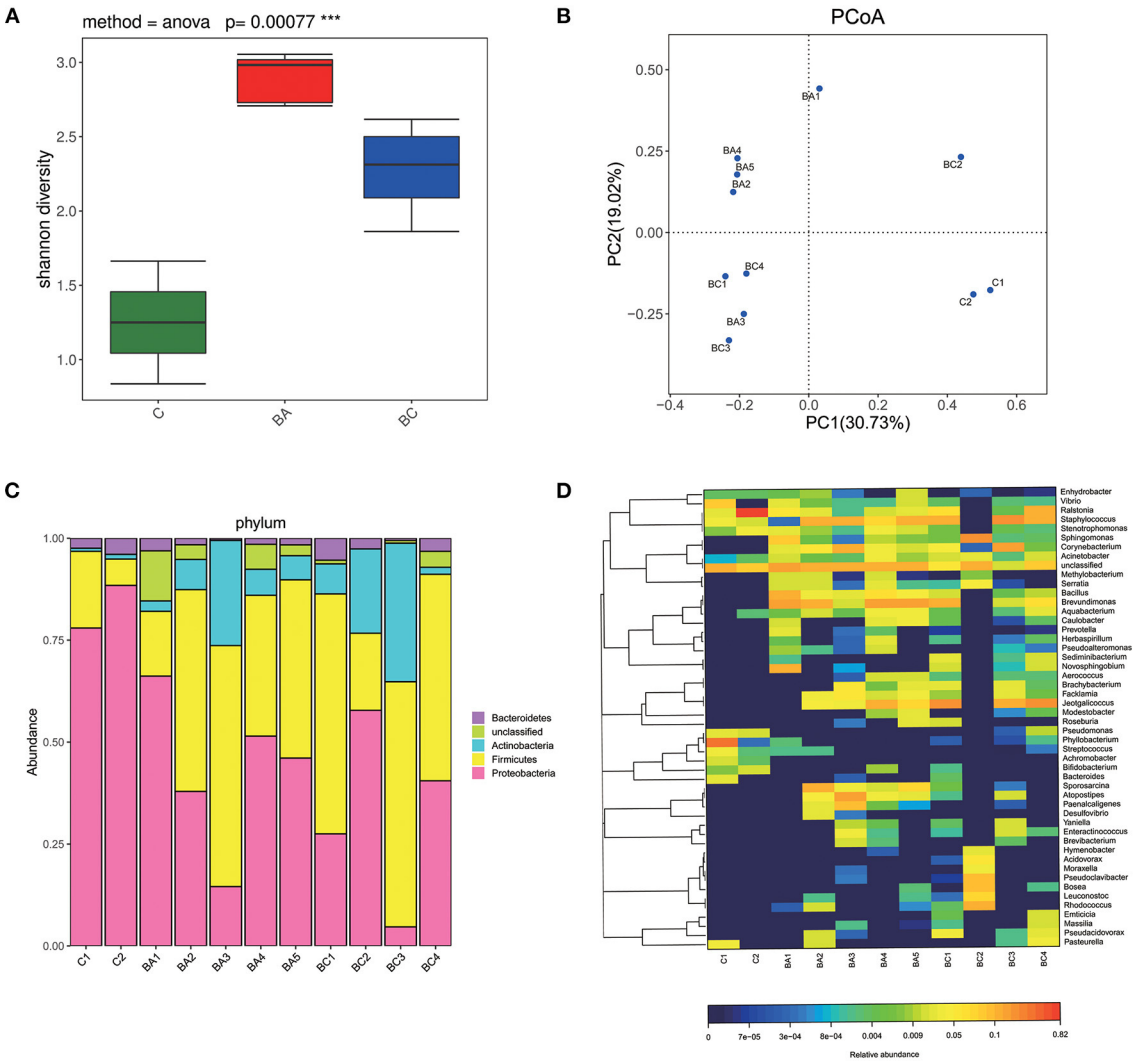
Bacterial composition and diversity. **(A)** Alpha diversity was significantly different across three groups (*P* < 0.001). The C group had the least species of microbiome and the BA group had the largest. **(B)** Samples of each group were located closely on the PCoA plots. **(C)** The composition of microbial communities at phylum level. **(D)** The composition of microbial communities at genus level.

A total of 391,417 of 16S rRNA effective raw reads were generated from the 11 samples, and the clean raw reads optimized after were 138,352, Supplementary Table 1). The sequence depth per group varied, with the highest number of clean raw reads obtained from BA group (mean = 14881.4) and BC group (mean = 14965.25). C group obtained the least clean raw reads (mean = 2,042) (Supplementary Table 1). These reads were assigned to 127 OTUs. Alpha-diversity was calculated by Shannon index according to obtained reads and OTUs as shown in Figure 3A. The BA group had the richest bacterial diversity, followed by the BC group. The C group has the smallest Shannon index, suggesting a comparatively simple microbial community in this group. Significant differences in the alpha-diversity were identified across the three groups (*p* = 0.00077). Principal coordinate analysis indicated that samples within the same group were much more likely located closely on the PCoA plots (Figure 3B). Specifically, microbial communities in the uterine cavity of CBA/J mice were distinct before and during birth. Compared with the non-pregnant stage, pregnant CBA/J mice had more abundant microbial populations, and the degree of diversity was more significant when combined with DBA/2 mice. The intra-group differences in the three groups of samples were small.

### Characteristics of Intrauterine Microbiome in CBA/J Mice

*Proteobacteria* was the most dominant phylum in C group (mean = 83.21%) and BA group (mean = 43.23%) (Figure 3C). *Firmicutes* was dominant in BC group (mean = 46.4%) and the second dominant in both C group (mean = 12.63%) and BA group (mean = 40.55%). Figure 3D showed a heat map of relative abundances of OTUs at the genus level. *Ralstonia* was the most abundant in C group (mean = 42.21%). *Phyllobacterium, Staphylococcus* as well as *Vibrio* and *Pseudomonas* were typical taxa. *Acinetobacter, Aquabacterium, Sphingomonas, Pasteurella*, and *Achromobacte*r were also observed but at relatively low abundances of the total population. BA group had an abundance of *Brevundimonas* (mean = 16.5%) followed by *Staphylococcus, Corynebacterium, Bacillus*, and *Jeotgalicoccus*. In BC group, the most dominant genus was *Jeotgalicoccus* (mean = 21.05%) and the second dominant one was *Staphylococcus* (mean = 17.51%). *Corynebacterium, Sphingomonas, Ralstonia*, and *Brevundimonas* were also found at lower relative abundances.

**Figure 4:**
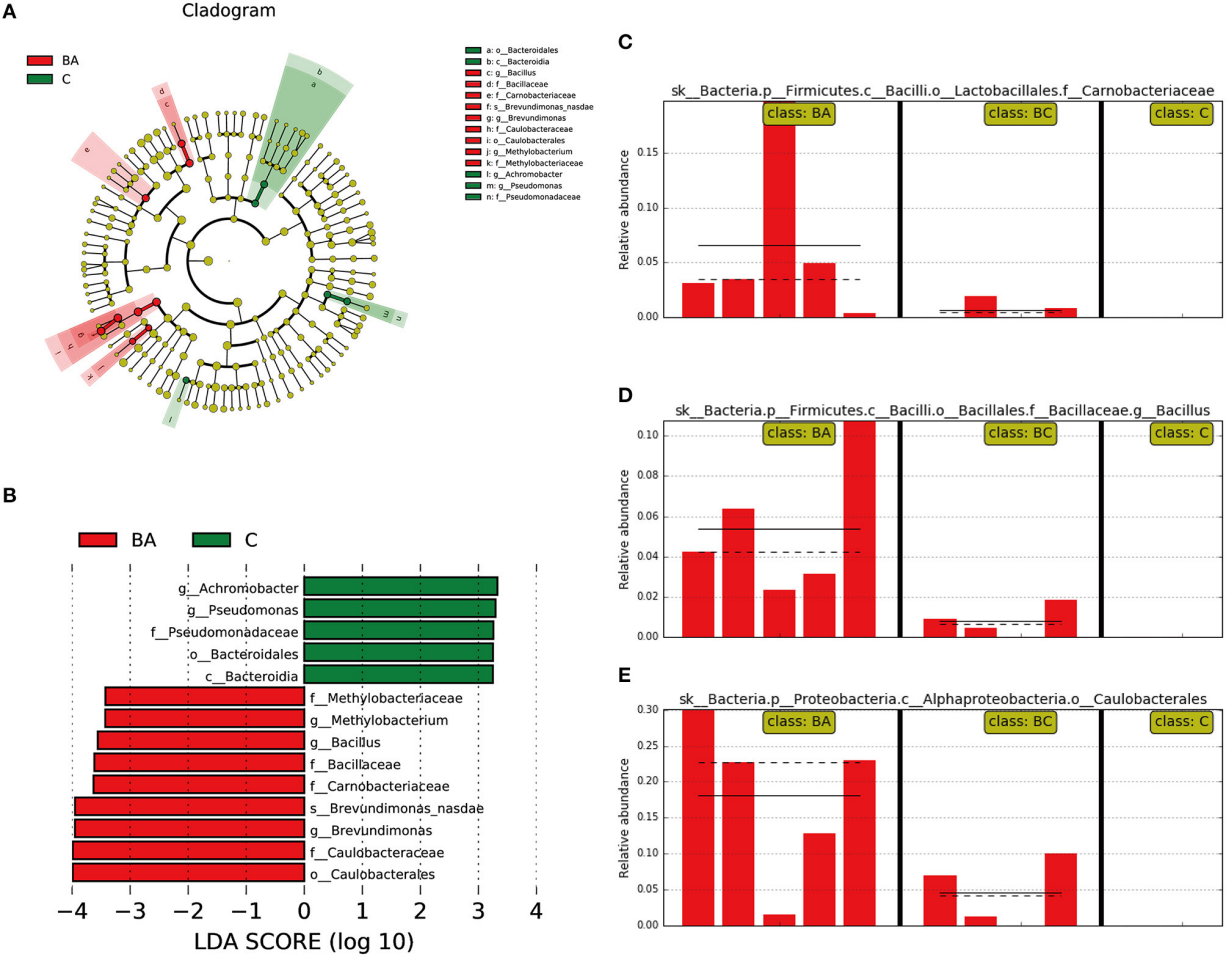
Different abundant microbiome among three groups. **(A,B)** *Achromobacter, Pseudomonas* and *Bacteroidales* as discriminatory features for C group; *Methylobacterium, Bacillus, Brevundimonas* and *Caulobacteraceae* were associated with BA group. **(C–E)** In comparison with BA group, C and BC group was more likely to have lower abundance or absence of *Carnobacteriaceae, Bacillus*, and *Caulobacterales*.

We then used LEfSe to classify various abundant microbiomes among three classes and found that *Achromobacter*, Pseudomonas and Bacteroidales were associated with discriminatory features in the C group, and *Methylobacterium, Bacillus, Brevundimonas* and *Caulobacteraceae* were associated with discriminatory features in group BA (Figures 4A,B). Comparing with BA group, C and BC group were more likely to have lower abundance or absence of *Carnobacteriaceae* (Figure 4C), *Bacillus* (Figure 4D), and *Caulobacterales* (Figure 4E).

### Functions of Intrauterine Microbiome in CBA/J Mice

**Figure 5:**
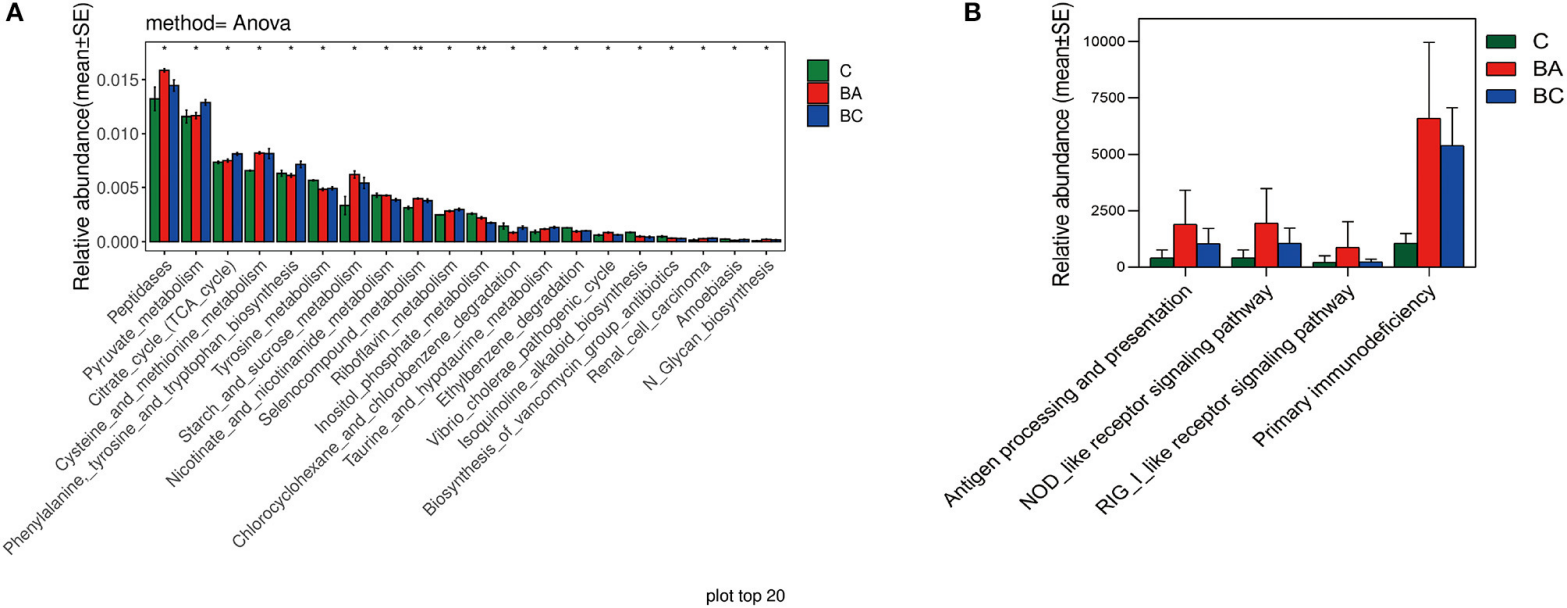
Functions of intrauterine microbiome in CBA/J mice. **(A)** The top 20 most different abundant enriched KEGG pathways. **(B)** The difference abundance enrichment of immune system between three groups.

The different abundance of microbiota was primarily enriched in metabolic pathways such as Peptidases, Pyruvate metabolism and TCA cycle, through the PICRUST and KEGG enrichment study (Figure 5A). Regarding the function and role of microbial communities in the immune system, there were no difference identified in NOD-like receptor signaling pathway across three groups (*p* = 0.295, Figure 5B).

## Discussion

To our knowledge, this is the first study to detect the microbiota in uterine cavity of CBA/J mice. Most research have focused on the composition of microbial communities in the human uterine cavity, amniotic fluid, placenta, and the downstream direction of causes of immune imbalance during pregnancy at the maternal-fetal interface. However, it is still necessary to clarify the composition, function, and relationship of microbial communities in the uterine cavity of CBA/J mice as well as possible mechanisms of the associated immune imbalance. Here we utilize the 16S rRNA sequencing technology and PICRUST enrichment to provide additional insights on this topic. In particular, we found that the compositions of microbial communities in pregnant uterine cavity of CBA/J mice were significantly distinct from those in unpregnant ones. When the mice were unpregnant, the diversity and the abundance of microbial communities were significantly lower. When the CBA/J mice mated with different kinds of male mice, there were variations in the uterine microbial populations as well. Semen has to liquefy in the vagina before reaching the uterine cavity after passing through the cervix, so that the egg can be fertilized. In this process, the microbiota from the cervix and vagina, as well as those from the male mice may enter the uterine cavity and colonized (10). Our finding suggest that the uterine cavity microbiome may be affected by the lower reproductive tract microbial communities and the semen of male mice. *Proteobacteria* is reported to be one of the major bacterial genera significantly enriched in the endometrial cancer cohort (11). It is the dominant phylum in both samples taken from the vaginas and uteri from giant pandas and may be attributed to pregnancy outcomes (12). During pregnancy, fat and vitamin intakes could increase the abundance of *Proteobacteria*, which is a pro-inflammatory maternal microbiota (13). Tapiainen et al. showed that the most abundant phyla were *Firmicutes* followed by *Proteobacteria* in first-pass meconium of newborn infants, and they were affected by maternal factors during pregnancy implying that they were transferred from the uterus (14). In our study, we found that *Proteobacteria* was the most dominant phylum in both unpregnant CBA/J mice and CBA/J×DBA/2 abortion-prone mice models. In CBA/J×BALB/C pregnant mice, the dominant phylum was *Firmicutes*. The abundance changes of *Proteobacteria*/*Firmicutes* may be a leading factor of abortion-prone in mice model.

Furthermore, we compared the distribution of microbial communities across three groups. Interestingly, *Carnobacteriaceae, Bacillus*, and *Caulobacterales* were more abundant than the usual pregnant classes in the abortion-prone model of mice whereas lacking in the unpregnant mice. *Carnobacteriaceae* may be attributed to hormone metabolites (15), which was increased in oral and gut samples after re-initiation of oral food intake after stroke (16). *Bacillus* was a spore-forming bacterium belonging to *Firmicutes* phylum, which can be activated in a suitable environment. *Bacillus* can be found in gastrointestinal tract of human and animals and stimulate the immune systems (17, 18). *Caulobacterales* belonging to *Proteobacteria* phylum can be obtained from soil and plants (19, 20). Future work should investigate whether they were the key bacteria leading to abortion and whether they can be used as biomarkers with potentially great clinical significance.

A normal pregnancy cannot be sustained without immune tolerance. The production of immune tolerance requires microbial colonization, therefore germ-free animals cannot achieve immune tolerance (21). However, when harmful microbial gains access to the uterine cavity, they can result in inflammation and disrupt the immune balance, eventually leading to abortion (22). We found the serum levels of IL-10, a representation of Th1 cytokines, were significantly lower in abortion-prone mice model whereas the serum level of TNF-α and INF-γ, a representation of Th2 cytokines, were significantly higher. This was consistent with previous studies (22–24). Regarding the role of microbial communities, we found that they can influence immune response through the NOD-like receptor signaling pathway, potentially because microbiome distribution changes could bind to the NOD-like receptor and participate in the Th1/Th2 immune balance bias that leads to abortion. Lastly, the following limitations should be considered. Samples were obtained from only uterine cavity, and we did not obtain the semen samples from different kinds of male mice, thus the composition of microbial communities among the three groups remain unclear. Besides, our data showed that microbial communities' functions were related to the signaling pathway of the NOD-like receptor, but the specific receptor remains unknown.

## Conclusion

Our study explored the composition of microbial communities in uterine cavity of CBA/J mice at both non-pregnant stage and pregnant stage, mated with BALB/c male mice and DBA/2 male mice. The differentially abundant microbiome may be attributed to immune tolerance through binding to NOD-like receptor. The specific microbiota in the uterine cavity that could be characterized as the biomarker of abortion-prone bacteria and how it affects the immune response remains to be elucidated in future work.

## Data Availability Statement

The original contributions presented in the study are included in the article/Supplementary Material, further inquiries can be directed to the corresponding authors.

## Ethics Statement

The animal study was reviewed and approved by All animal experimental protocols were approved by the Animal Ethics Committee of Sun Yat-Sen University (Guangzhou, China).

## Author Contributions

We declare that all the listed authors have participated actively in the study and all meet the requirements of the authorship. HC designed the study and wrote the protocol. SB and BH performed research. SF contributed important reagents. LZ and MC managed the literature searches and analyses. MZ and LH undertook the statistical analysis. JT, JZ, and ZZ wrote the first draft of the manuscript. ZZ made a contribution to the recent revision for this manuscript. All authors approved the final version of the manuscript.

## Conflict of Interest

The authors declare that the research was conducted in the absence of any commercial or financial relationships that could be construed as a potential conflict of interest.
