# Supplementary material for: Changes in the Distribution of Intrauterine Microbiota May Attribute to Immune Imbalance in the CBA/J×DBA/2 Abortion-Prone Mice Model
Source: Front Immunol. 2021 Mar 8;12:641281. doi: 10.3389/fimmu.2021.641281 (PMC7982683; doi:10.3389/fimmu.2021.641281)
Supplement: Supplementary file 1 [file Table_1.DOCX]

**Table S1. Dataset features of all samples**

| Samples | Effective raw reads | Clean raw reads |
| --- | --- | --- |
| BA1 | 25879 | 10004 |
| BA2 | 27016 | 14843 |
| BA3 | 29751 | 18580 |
| BA4 | 36059 | 14470 |
| BA5 | 37164 | 16510 |
| BC1 | 33324 | 16169 |
| BC2 | 48871 | 4058 |
| BC3 | 28427 | 22624 |
| BC4 | 40606 | 17010 |
| C1 | 36556 | 1308 |
| C2 | 47764 | 2776 |
